# Impact of hepatic function on serum procalcitonin for the diagnosis of bacterial infections in patients with chronic liver disease: A retrospective analysis of 324 cases

**DOI:** 10.1097/MD.0000000000004270

**Published:** 2016-07-29

**Authors:** Junyan Qu, Ping Feng, Yan Luo, Xiaoju Lü

**Affiliations:** a Center of Infectious Disease; b Department of Rheumatology, West China Hospital, Sichuan University, Chengdu, China.

**Keywords:** chronic liver disease, infection, procalcitonin, total bilirubin

## Abstract

Although procalcitonin (PCT) is a valid marker for early diagnosis of bacterial infections, it is unclear whether its accuracy in predicting bacterial infections is affected by impaired liver function. This study aimed to assess the impact of compromised liver function on the diagnostic value of PCT.

This retrospective study was conducted between January 2013 and May 2015. A total of 324 patients with chronic liver disease were enrolled. Routine laboratory measurements and PCT were performed. Patients were divided into 3 groups according to clinical diagnosis: chronic hepatitis (group 1), decompensated cirrhosis (group 2), and acute-on-chronic liver failure/chronic liver failure (group 3). The correlation between PCT and liver function was analyzed. The area under the receiver operating characteristic (AUCROC) curve of PCT was analyzed according to infection status and liver function.

PCT was more accurate than white blood cell count (*P* < 0.001) and percentage of neutrophils (*P* < 0.001) in detecting bacterial infections in patients with impaired liver function. In patients without infection, PCT had a moderate positive correlation with serum total bilirubin (TBIL) (*r* = 0.592), and a weak correlation with model for end-stage liver disease score (*r* = 0.483) and international normalized ratio (*r* = 0.389). The AUCROC and optimum thresholds of PCT and for predicting bacterial infections at different levels of TBIL were 0.907 (95% CI 0.828–0.958) and 0.38 ng/mL, respectively, for TBIL <5 mg/dL, 0.927 (95% CI 0.844–0.974) and 0.54 ng/mL (5 mg/dL ≤TBIL<10 mg/dL), 0.914 (95% CI 0.820–0.968) and 0.61 ng/mL (10 mg/dL ≤TBIL<20 mg/dL), 0.906 (95% CI 0.826–0.958) and 0.94 ng/mL (TBIL ≥20 mg/dL), respectively.

This study demonstrated that PCT was a valuable marker of bacterial infection in patients with chronic liver diseases. TBIL affected PCT threshold, so different cut-offs should be used according to different TBIL values.

## Abstract 2

Supplemental Digital Content is available in the text

## 1 Introduction

Chronic liver disease can result in cirrhosis and hepatic carcinoma, and is thus a considerable burden particularly in developing countries. The main causes of chronic liver disease are hepatitis B (HBV) and C (HCV) virus infections, alcohol, and metabolic fatty liver disease.^[1]^ Patients with either acute or chronic liver disease are more likely to have concurrent infections due to innate immune dysfunction, genetic predisposition, and intrinsic cellular defects, which in turn leads to impaired liver function and ultimately higher mortality.^[2]^ Spontaneous bacterial peritonitis (SBP) is the most common type of infection in cirrhosis patients with decompensated hepatic function, followed by urinary tract infections and pneumonia.^[3,4]^ Diagnosis of bacterial infections is difficult in those patients without typical manifestations and appropriate diagnostic tools. Leukocyte count has limited value because patients with chronic liver disease often have deficient neutrophil recruitment due to pancytopenia caused by accompanying hypersplenism.^[5]^ Positive bacteria culture is regarded as a gold standard in diagnosing infections, but is time-consuming and the positive rate is low. Around 60% of patients with decompensated cirrhosis have negative ascites bacterial culture results even where SBP has been diagnosed based on clinical manifestations and other laboratory examinations.^[6]^ Thus, a reliable method of early identification of bacterial infections is warranted to improve prognosis for these patients.

Procalcitonin (PCT) is a calcitonin precursor hormone, and the serum concentration in healthy people is <0.01 ng/mL. Serum PCT is raised in bacterial infections, but remains low in viral infections and nonspecific inflammatory diseases.^[7]^ Many studies showed that PCT was a helpful biomarker in detecting bacterial infections.^[8]^ PCT-guided antibiotic stewardship in persistent bacterial infections may reduce the duration of antibiotic therapy.^[9]^ Liver is one of the tissues that produce PCT in response to bacterial infections,^[10]^ leading to speculation as to whether PCT levels would be lower in patients with impaired liver function. Aside from that, little is known about the elimination pathways of PCT, although it is likely that liver function interferes with PCT concentrations. Research led by Bota indicated that the serum PCT level was not significantly lower in patients with hepatic cirrhosis than in other patients without cirrhosis.^[11]^ A few studies have evaluated the diagnostic value of PCT in patients with liver diseases, and some of these suggested that PCT was not an accurate marker of SBP in cirrhotic patients,^[12]^ and the cut-off values for prediction of infections were highly variable.^[13–16]^ However, no study has assessed the relationship between decreased liver function and serum PCT concentration. We postulated that the role of PCT in diagnosis of bacterial infections may be affected by liver dysfunction, so it would be inappropriate to use the same cut-off value in the patients with compromised liver function in different degrees.

To assess this hypothesis, we investigated the influence of liver function on the diagnostic accuracy of PCT in patients with chronic liver disease. We also assessed the optimal cut-off values of PCT for early detection of bacterial infections in these patients according to different degrees of liver dysfunction.

## 2 Materials and methods

### 2.1 Study population

The retrospective study was conducted between January 2013 and May 2015 at the Center of Infectious Diseases, West China Hospital, Sichuan University, China (a 4300-bed academic tertiary care hospital). The protocol for this research was approved by the Ethics Committee of West China Hospital, Sichuan University, who waived the need for consent because the study was retrospective and data were to be analyzed anonymously.

All the adult patients (≥18 years old) with chronic liver disease admitted to the ward of the Center of Infectious Diseases were enrolled. Exclusion criteria were age under 18 years, pregnancy, thyroid tumor, moderate and severe renal insufficiency (creatinine clearance <50 mL min^–1^ per 1.73 m^2^), acute liver disease, and other severe comorbidities such as neoplastic, cardiac and hematologic diseases, and fungal infections.

Chronic hepatitis was defined as inflammation of the liver that continued without improvement for ≥6 months. The diagnosis of liver cirrhosis was made according to clinical and laboratory criteria without histological confirmation. The causes of cirrhosis included chronic HBV and/or HCV infection, alcoholism, autoimmune hepatitis, and cryptogenic causes according to the medical records. The severity of liver cirrhosis and liver failure were evaluated by Child–Turcotte–Pugh (CTP) score and model for end–stage liver disease (MELD) score. SBP was defined as ascitic fluid infections with positive ascitic fluid bacterial culture and/or an elevated ascitic fluid absolute polymorphonuclear leukocyte count (≥250 cells/mm^3^), from which intra-abdominal surgical intervention as a suspicious source of infection was excluded. Definitive diagnosis of infection was determined by medical history, clinical manifestations, physical examinations, and laboratory and imaging tests. Pneumonia was diagnosed when a positive sputum culture and/or a typical chest x-ray or computerized tomography were found. Bloodstream infection was diagnosed from positive blood culture. Urinary tract infection was considered when bacterial colony counts were higher than 10^5^/mL in a mid-stream “clean catch” urine and and/or a positive urine culture. Criteria for decompensation in cirrhosis patients were: ascites, jaundice, encephalopathy or variceal hemorrhage, with international normalized ratio (INR) <1.5. Acute-on-chronic liver failure referred to acute hepatic insult manifesting as jaundice (serum total bilirubin ≥5 mg/dL) and coagulopathy (INR ≥1.5), complicated by ascites and/or encephalopathy within 4 weeks in patients with a history of chronic liver disease.^[17]^ Chronic liver failure was defined as liver function decompensation based on liver cirrhosis, with similar clinical manifestations to acute-on-chronic liver failure. INR ≥1.5 in this study was important for definitive diagnosis of liver failure and decompensated cirrhosis. Patients were divided into 3 groups according to clinical diagnosis (group 1: chronic hepatitis; group 2: decompensated cirrhosis; group 3: acute-on-chronic liver failure/chronic liver failure). Each group was then divided into 2 subgroups (infection group and noninfection group) based on infection status.

### 2.2 Measurement

We reviewed the medical records of patients involved in this study using data exclusively from electronic medical records of our hospital verified independently by 2 authors. Routine laboratory tests such as a complete blood count and a blood biochemical test were performed. All samples (including blood, sputum, ascites, urine) were collected under sterile conditions and blood samples for bacterial culture were collected in >2 sets. The serum PCT level was measured on admission using an electrochemiluminescence immunoassay B.R.A.H.M.S. PCT ELECSYS^®^ (Roche Elecsys E170, Roche Diagnostics GmbH Mannheim, Germany). This assay is more sensitive^[18]^ than others and has a detection limit of 0.02 ng/mL.

### 2.3 Statistical analysis

Data were analyzed using SPSS version 17.0 software package (SPSS Inc., Chicago, IL). A normality test was performed for all quantitative variables. Continuous variables were presented as median and interquartile range. Nonparametric statistical methods were used for non-normally distributed data. Correlations were assessed by Spearman's test. The correlation coefficient, *r*, was used to measure the strength and the direction of a linear relationship between 2 variables. A correlation >0.8 was generally described as strong, whereas 0.2 <*r*< 0.5 was described as weak correlation. Significance testing was carried out using the T-test or Mann–Whitney *U* test. Receiver operating characteristic (ROC) curves were analyzed using MedCalc software version 11.5.1.0 (MedCalc Software bvba, Ostend, Belgium). The area under the curve (AUC) was calculated to evaluate diagnostic accuracy. ROC curve analysis was carried out using the method described by DeLong et al.^[19]^ A 2-tailed *P* < 0.05 was considered significant.

## 3 Results

A total of 324 patients (mean age 45.19 ± 12.76 years; 259 male) with chronic liver disease were included in this study. In group 1, 5 patients (7.14%) had an infection and 65 patients (92.86%) did not; in group 2, 52 patients had infections (38.81%) and 82 patients (61.19%) did not; and in group 3, 68 patients (56.67%) had infections and 52 patients (43.33%) did not. The demographic and laboratory characteristics of the included patients are listed in Table 1. There were no significant differences in age and sex between the groups.

**Table 1:**
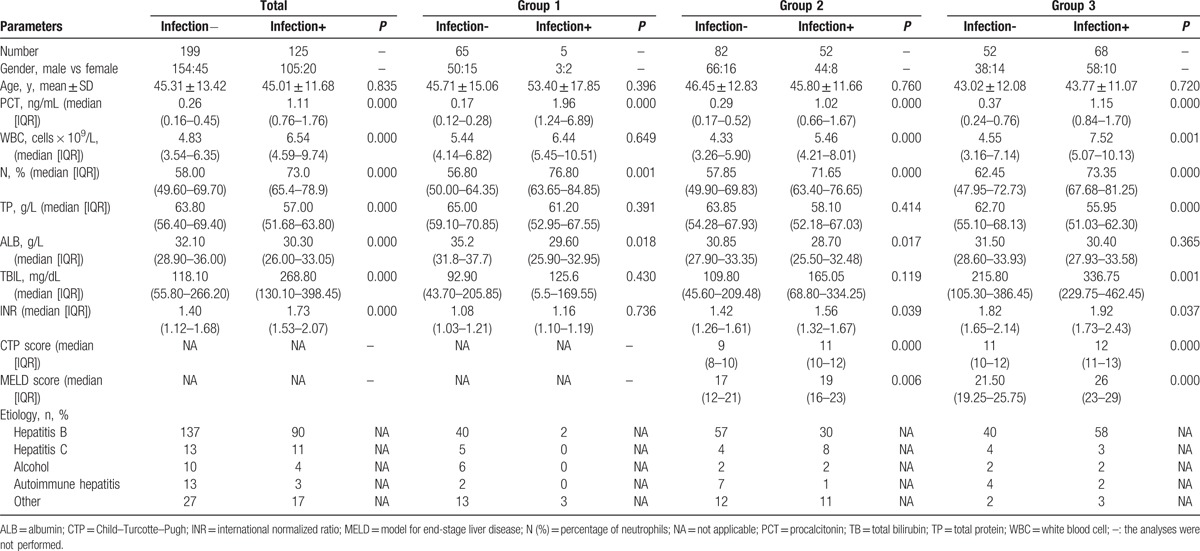
Demographic and laboratory characteristics of enrolled patients (n = 324) with liver diseases.

On admission, 61.42% (199/324) of patients were free of infection and the remaining 38.58% (125/324) had an infection. SBP was the most common type of infection (73/125, 58.4%), followed by pulmonary infection (46/125, 37.6%). One patient of the 125 (0.80%) had clinical evidence of infection but the infectious site could not be determined despite comprehensive investigation. Twenty-three cases had multisite infections. Ninety-one clinical specimens were collected. Of the specimens, positive culture results were obtained from 8 (17.4%) of 46 ascitic fluid samples, and 12 (41.3%) of 29 qualified sputum samples. There were only 29/91 cases (31.8%) with positive culture results. Identified pathogens are listed in Table 2. Most infections were caused by Gram-negative bacteria (23/29, 79.3%), predominantly *Klebsiella pneumoniae* (10/29, 34.5%,) followed by *Escherichia coli* (6/29, 20.7%).

**Table 2:**
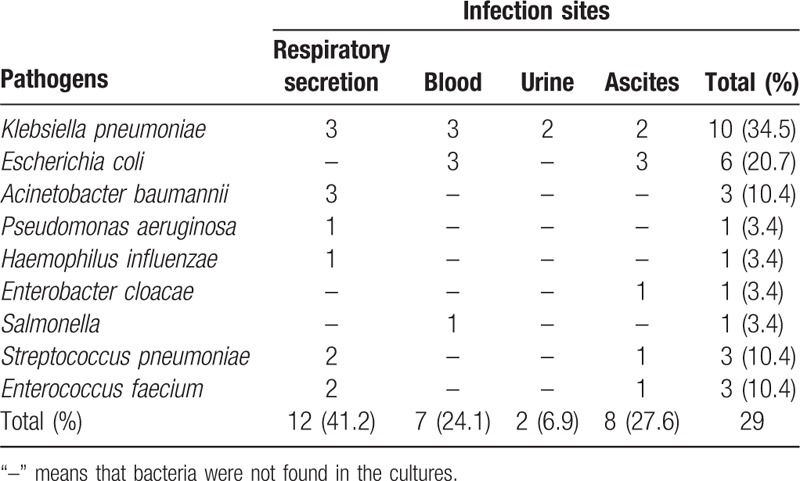
Pathogens identified in the hepatopathy patients with positive culture.

**Figure 1:**
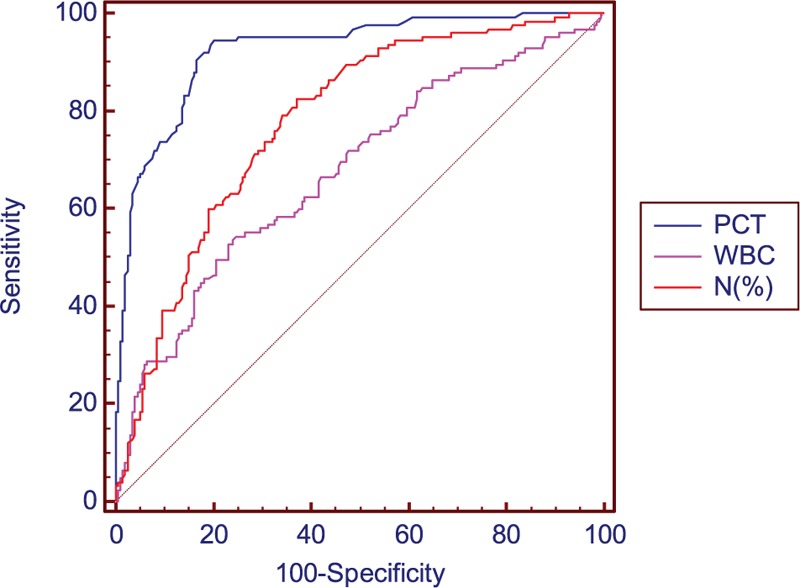
Receiver operating characteristic (ROC) curves for PCT, WBC, and percentage of neutrophils for diagnosis of infection in patients with chronic liver diseases. Area under curves were 0.923 (95% CI 0.889–0.950), 0.676 (95% CI 0.622–0.727), and 0.778 (95% CI 0.729–0.822) for PCT, WBC, and percentage of neutrophils, respectively. CI = confidence interval, PCT = procalcitonin, ROC = receiver operating characteristic, WBC = white blood cell.

Figure 1 depicts the ROC curves of PCT, white blood cell count (WBC) and proportion of neutrophils for infection discrimination in patients with chronic liver disease. The most effective cut-off value of PCT for diagnosing infection in patients with impaired liver function was 0.53 ng/mL. AUC values for these parameters are listed in Table 3. PCT was more effective than WBC (*P* < 0.001) and percentage of neutrophils (*P* < 0.001) for detecting bacterial infections. The AUC for the diagnosis of infection was 0.923 (95% CI 0.889–0.950) for PCT, and 0.778 (95% CI 0.729–0.822) for proportion of neutrophils. In peripheral blood, WBC ≥10 × 10^9^/L and percentage of neutrophils ≥75% were used as a cut-off to identify infections in patients with impaired liver function, for which Youden's index was 17.58% and 27.12%, respectively. The diagnostic efficiency decreased.

**Table 3:**
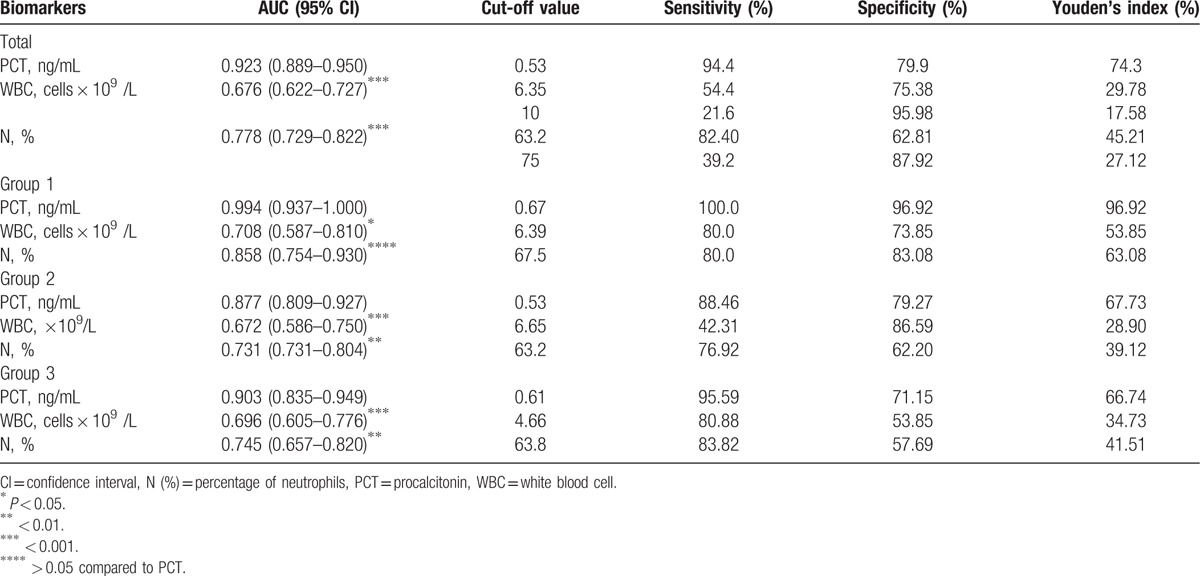
Area under the curves (AUC) of the receiver operating characteristic for procalcitonin (PCT), leukocyte count (WBC), percentage of neutrophils and the best cut-off values to detect bacterial infection from patients with liver disease.

**Figure 2:**
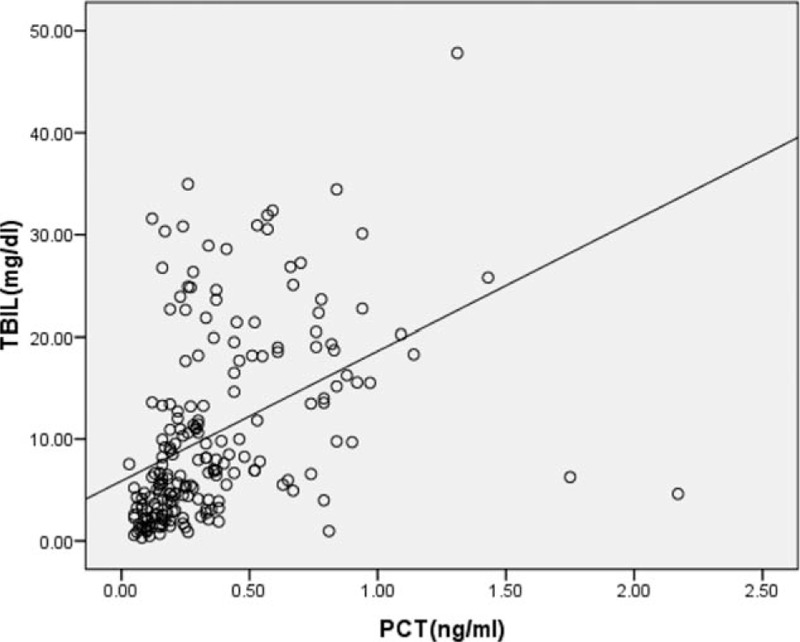
Association between procalcitonin (PCT) levels and total bilirubin (TBIL) in patients with impaired liver function. Spearman correlation analysis was used to obtain *r* and *P* values. PCT was significantly correlated with TBIL (*r* = 0.592, *P* = 0.000). PCT = procalcitonin, TBIL = total bilirubin.

The correlation between PCT and liver function was analyzed in patients without infection (Table 4). Patients without infection in group 1 were excluded when analyzing the correlation between PCT and CTP score or MELD score. PCT had a moderate correlation with total bilirubin (TBIL) (*r* = 0.592), and a weak correlation with MELD score (*r* = 0.483) and INR (*r* = 0.389) (Fig. 2).

**Table 4:**
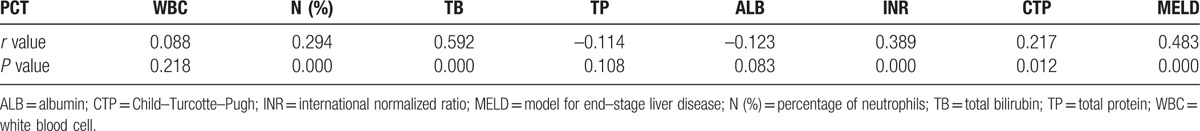
The correlation between procalcitonin (PCT) and liver function.

Patients were divided into 4 groups based on TBIL (group A: TBIL <5 mg/dL; group B: 5 mg/dL ≤TBIL<10 mg/dL; group C: 10 mg/dL ≤TBIL<20 mg/dL; group D: TBIL ≥20 mg/dL). The ROC curves of PCT for diagnosing infection in patients with different serum TBIL levels are presented in Figure S1 in the Supplementary Appendix. The AUC of PCT and the most accurate cut-off values in the 4 groups with distinct TBIL levels are listed in Table 5. The most accurate cut-off values of PCT for diagnosis of infections were 0.38 ng/mL (group A), 0.54 ng/mL (group B), 0.61 ng/mL (group C), and 0.94 ng/mL (group D). It is widely accepted that 0.25 ng/mL for PCT is the cut-off value for lower respiratory tract infections in the general population,^[20–21]^ so when using 0.25 ng/mL to identify infectious complications in patients with chronic liver disease (Table 5), the diagnostic sensitivity improved but the specificity decreased significantly, and thus the diagnostic efficiency also decreased.

**Table 5:**
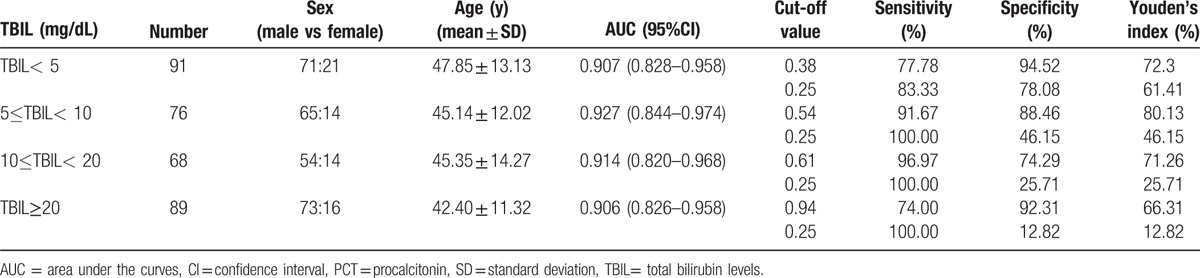
Area under the curves (AUC) for procalcitonin (PCT) and the best cut-off values to detect bacterial infection from patients with liver disease by total bilirubin levels (TBIL).

## 4 Discussion

Early identification of bacterial infections in patients with liver disease is important. Many previous studies have shown that PCT was superior to C-reactive protein, interleukin-6 and leukocyte counts in early diagnosis of bacterial infections in patients without liver disease.^[22–24]^ PCT was also a valuable marker of bacterial infections in different clinical situations, such as pneumonia, severe sepsis/septic shock, and bloodstream infection. However, applying PCT to abdominal infections, a common complication of liver disease, remains controversial.^[25]^ Little is known about whether impaired liver function could impact the diagnostic value of PCT in patients with liver disease.

In this study, we found that the serum level of PCT and percentage of neutrophils were significantly increased in bacterial infection groups regardless of the severity of liver disease. The PCT levels showed the best diagnostic accuracy for predicting bacterial infections in the ROC curve analysis, followed by percentage of neutrophils and peripheral blood leukocyte count. This suggests that PCT is a valuable biomarker in detecting bacterial infections in patients with liver disease, which is consistent with the findings of previous studies.^[13–16]^ The leukocyte count of group 1 was increased in patients with bacterial infections, although the difference was not significant, which may be related to the limited number of cases.

The results of this study show that the serum PCT level with a cut-off value of 0.53 ng/mL had the best specificity and sensitivity to identify bacterial infections in patients with liver disease. Although most previous studies showed that PCT was a validated biomarker of infections in patients with liver disease, there was considerable discrepancy in the optimal cut-offs.^[13–16]^ This prominent variation could be explained by differences in methodology (such as different inclusion and exclusion criteria, different baselines of liver function, different etiologies and different races), the severity of infectious episodes and the relatively small number of cases involved. A recent study demonstrated that a cut-off between 0.1 and 0.5 ng/mL was applicable in patients with lower respiratory tract infections.^[26]^ Another meta–analysis indicated that a cut-off between 1.0 and 2.0 ng/mL was helpful for identifying patients with sepsis.^[27]^ Therefore, the optimal cut-off values for PCT to detect infection may be related to different organ function status, different types, and sites of infections. Liver dysfunction may affect the accuracy of PCT to identify bacterial infections.

In our study, we found the most common bacterial infections were SBP and pneumonia. Gram-negative bacteria, such as *E coli* and *Klebsiella spp*, were the main pathogens. Similar to previous reports, the most common Gram-positive bacteria were *Pneumococcus* and *Enterococcus faecium*.^[28–29]^ Bacterial translocation is the key step in the pathogenesis of SBP in cirrhotic patients. Gram-negative enteric bacteria, *enterococci* and other *streptococci*, are the bacteria most easily translocated to mesenteric lymph nodes.^[30]^ Only 29 bacterial strains were isolated, perhaps because our hospital is a large tertiary referral hospital, so infectious complications in some patients were diagnosed and antibacterial drugs used empirically before they were admitted to our hospital, so the rate of positive culture was low.

Our study demonstrated a moderately significant correlation between PCT and TBIL in noninfected patients. This result implies that PCT may be influenced by liver function, so this should be taken into account when using PCT to diagnose infection. This may partly explain why PCT seemed to be an inaccurate marker of SBP in patients with liver disease and the cut-off values for identifying infections were highly variable in different studies.^[12–16]^ PCT is secreted by adherent monocytes and macrophage-activated adipocytes in inflammation.^[31]^ Liver cells are damaged in liver diseases, whereas monocytes and adipocytes are not. In addition, endotoxin levels increase in liver diseases and are associated with a serious degree of liver disease. Endotoxin plays an important role in increased levels of PCT.^[32]^ This may partly explain why PCT had a positive correlation with TBIL. A recently published study indicated that in patients with various forms of acute liver failure, PCT appeared to correlate with aminotransferase levels.^[33]^ That study included patients with acute liver failure, who often have higher alanine aminotransferase (ALT), aspartate aminotransferase (AST), and endotoxin levels in the early stage than in chronic liver disease. The high ALT or ALT levels may appear to have been the cause of the high PCT levels in these patients, but high endotoxin level is probably the major reason. Different studies include subjects with different diseases and different phases of disease, which may yield different results. Besides, our study only showed a moderate correlation of PCT with TBIL, and there are likely other factors involved. Further research into this correlation is required.

The metabolic pathway of PCT remains unclear. One study showed that renal elimination was the major pathway for the clearance of PCT,^[34]^ whereas another study found a high level of PCT in patients with renal dysfunction without signs of infections.^[35]^ Yet another study indicated that the plasma clearance rate of PCT correlated weakly with renal function.^[36]^ The liver is an essential component of the body's metabolic system, and the bilirubin level may reflect severity of liver damage, meaning that the liver may be one location for elimination of PCT. This hypothesis requires further confirmation.

There are some limitations to our study. First, we assessed liver function in groups 2 and 3 using the CTP and MELD scores, but patients in group 1 were excluded when we analyzed the correlation between PCT and CTP or MELD scores. However, all patients were included when we analyzed the correlation between PCT and other indicators of liver function. This may have caused selection bias. Second, we only detected PCT levels at admission. Serial monitoring of PCT levels may be more helpful in guiding the application of antibiotics. Third, 70% of patients had hepatitis B virus related liver disease. There is a difference in the etiology of chronic liver disease between Western and Asian populations. Whether our findings in an Asian population may transfer to other populations needs to be evaluated in future multicenter studies.

## 5 Conclusions

We found that PCT was a helpful marker in identifying bacterial infections in patients with chronic liver disease. PCT had a moderate positive correlation with serum TBIL level, and a weak correlation with MELD score and INR. Serum TBIL influenced the PCT thresholds, so different cut-offs should be applied based on different TBIL levels. Although PCT was more accurate than other parameters for indicating infections, a patient's clinical manifestations, other laboratory tests, and imaging data should be integrated in order to make final decisions for diagnosis and treatment.

## Acknowledgments

The authors thank the nurses at the Center of Infectious Disease, West China Hospital, Sichuan University for clinical assistance.

## Supplementary Material

Supplemental Digital Content

## References

[1] Wang FS Fan JG Zhang Z The global burden of liver disease: the major impact of China. *Hepatology* 2014; 60:2099–2108. 25164003 10.1002/hep.27406 PMC4867229

[2] Lin KH Wang FL Wu MS Serum procalcitonin and C-reactive protein levels as markers of bacterial infection in patients with liver cirrhosis: a systematic review and meta-analysis. *Diagn Microbiol Infect Dis* 2014; 80:72–78. 24974271 10.1016/j.diagmicrobio.2014.03.029

[3] Jalan R Fernandez J Wiest R Bacterial infections in cirrhosis: a position statement based on the EASL Special Conference 2013. *J Hepatol* 2014; 60:1310–1324. 24530646 10.1016/j.jhep.2014.01.024

[4] Shalimar Acharya SK Difficult to treat spontaneous bacterial peritonitis. *Trop Gastroenterol* 2013; 34:7–13. 23923368 10.7869/tg.2012.84

[5] Fiuza C Salcedo M Clemente G In vivo neutrophil dysfunction in cirrhotic patients with advanced liver disease. *J Infect Dis* 2000; 182:526–533. 10915084 10.1086/315742

[6] Enomoto H Inoue S Matsuhisa A Diagnosis of spontaneous bacterial peritonitis and an in situ hybridization approach to detect an “unidentified” pathogen. *Int J Hepatol* 2014; 2014:634617. 25132996 10.1155/2014/634617 PMC4123576

[7] Limper M de Kruif MD Duits AJ The diagnostic role of procalcitonin and other biomarkers in discriminating infectious from non-infectious fever. *J Infect* 2010; 60:409–416. 20347867 10.1016/j.jinf.2010.03.016

[8] Wacker C Prkno A Brunkhorst FM Procalcitonin as a diagnostic marker for sepsis: a systematic review and meta-analysis. *Lancet Infect Dis* 2013; 13:426–435. 23375419 10.1016/S1473-3099(12)70323-7

[9] Christ-Crain M Jaccard-Stolz D Bingisser R Effect of procalcitonin-guided treatment on antibiotic use and outcome in lower respiratory tract infections: cluster-randomised, single-blinded intervention trial. *Lancet* 2004; 363:600–607. 14987884 10.1016/S0140-6736(04)15591-8

[10] Muller B White JC Nylen ES Ubiquitous expression of the calcitonin-i gene in multiple tissues in response to sepsis. *J Clin Endocrinol Metab* 2001; 86:396–404. 11232031 10.1210/jcem.86.1.7089

[11] Bota DP Van Nuffelen M Zakariah AN Serum levels of C-reactive protein and procalcitonin in critically ill patients with cirrhosis of the liver. *J Lab Clin Med* 2005; 146:347–351. 16310518 10.1016/j.lab.2005.08.005

[12] Spahr L Morard I Hadengue A Procalcitonin is not an accurate marker of spontaneous bacterial peritonitis in patients with cirrhosis. *Hepatogastroenterology* 2001; 48:502–505. 11379342

[13] Lazzarotto C Ronsoni MF Fayad L Acute phase proteins for the diagnosis of bacterial infection and prediction of mortality in acute complications of cirrhosis. *Ann Hepatol* 2013; 12:599–607. 23813138

[14] Elefsiniotis IS Skounakis M Vezali E Clinical significance of serum procalcitonin levels in patients with acute or chronic liver disease. *Eur J Gastroenterol Hepatol* 2006; 18:525–530. 16607149 10.1097/00042737-200605000-00012

[15] Li CH Yang RB Pang JH Procalcitonin as a biomarker for bacterial infections in patients with liver cirrhosis in the emergency department. *Acad Emerg Med* 2011; 18:121–126. 21276124 10.1111/j.1553-2712.2010.00991.x

[16] Cekin Y Cekin AH Duman A The role of serum procalcitonin levels in predicting ascitic fluid infection in hospitalized cirrhotic and non-cirrhotic patients. *Int J Med Sci* 2013; 10:1367–1374. 23983598 10.7150/ijms.6014 PMC3752724

[17] Sarin SK Kumar A Almeida JA Acute-on-chronic liver failure: consensus recommendations of the Asian Pacific Association for the study of the liver (APASL). *Hepatol Int* 2009; 3:269–282. 19669378 10.1007/s12072-008-9106-x PMC2712314

[18] de Wolf HK Gunnewiek JK Berk Y Comparison of a new procalcitonin assay from roche with the established method on the brahms kryptor. *Clin Chem* 2009; 55:1043–1044. 19264851 10.1373/clinchem.2008.117655

[19] DeLong ER DeLong DM Clarke-Pearson DL Comparing the areas under two or more correlated receiver operating characteristic curves: a nonparametric approach. *Biometrics* 1988; 44:837–845. 3203132

[20] Schuetz P Christ-Crain M Thomann R Effect of procalcitonin-based guidelines vs standard guidelines on antibiotic use in lower respiratory tract infections: the ProHOSP randomized controlled trial. *JAMA* 2009; 302:1059–1066. 19738090 10.1001/jama.2009.1297

[21] Schuetz P Albrich W Christ-Crain M Procalcitonin for guidance of antibiotic therapy. *Expert Rev Anti Infect Ther* 2010; 8:575–587. 20455686 10.1586/eri.10.25

[22] Lee CC Chen SY Tsai CL Prognostic value of mortality in emergency department sepsis score, procalcitonin, and C-reactive protein in patients with sepsis at the emergency department. *Shock* 2008; 29:322–327. 17724429 10.1097/shk.0b013e31815077ca

[23] Prat C Sancho JM Dominguez J Evaluation of procalcitonin, neopterin, C-reactive protein, IL-6 and IL-8 as a diagnostic marker of infection in patients with febrile neutropenia. *Leuk Lymphoma* 2008; 49:1752–1761. 18661397 10.1080/10428190802258956

[24] Yuan LY Ke ZQ Wang M Procalcitonin and C-reactive protein in the diagnosis and prediction of spontaneous bacterial peritonitis associated with chronic severe hepatitis B. *Ann Lab Med* 2013; 33:449–454. 24205495 10.3343/alm.2013.33.6.449 PMC3819445

[25] Schuetz P Albrich W Mueller B Procalcitonin for diagnosis of infection and guide to antibiotic decisions: past, present and future. *BMC Med* 2011; 9:107. 21936959 10.1186/1741-7015-9-107 PMC3186747

[26] Schuetz P Chiappa V Briel M Procalcitonin algorithms for antibiotic therapy decisions: a systematic review of randomized controlled trials and recommendations for clinical algorithms. *Arch Intern Med* 2011; 171:1322–1331. 21824946 10.1001/archinternmed.2011.318

[27] Schuetz P Briel M Christ-Crain M Procalcitonin to guide initiation and duration of antibiotic treatment in acute respiratory infections: an individual patient data meta-analysis. *Clin Infect Dis* 2012; 55:651–662. 22573847 10.1093/cid/cis464 PMC3412690

[28] Fernandez J Navasa M Gomez J Bacterial infections in cirrhosis: epidemiological changes with invasive procedures and norfloxacin prophylaxis. *Hepatology* 2002; 35:140–148. 11786970 10.1053/jhep.2002.30082

[29] Brann OS Infectious complications of cirrhosis. *Curr Gastroenterol Rep* 2001; 3:285–292. 11469997 10.1007/s11894-001-0051-2

[30] Steffen EK Berg RD Deitch EA Comparison of translocation rates of various indigenous bacteria from the gastrointestinal tract to the mesenteric lymph node. *J Infect Dis* 1988; 157:1032–1038. 3283254 10.1093/infdis/157.5.1032

[31] Linscheid P Seboek D Schaer DJ Expression and secretion of procalcitonin and calcitonin gene-related peptide by adherent monocytes and by macrophage-activated adipocytes. *Crit Care Med* 2004; 32:1715–1721. 15286549 10.1097/01.ccm.0000134404.63292.71

[32] Dandona P Nix D Wilson MF Procalcitonin increase after endotoxin injection in normal subjects. *J Clin Endocrinol Metab* 1994; 79:1605–1608. 7989463 10.1210/jcem.79.6.7989463

[33] Rule JA Hynan LS Attar N Procalcitonin identifies cell injury, not bacterial infection, in acute liver failure. *PLoS One* 2015; 10:e0138566 doi: 10.1371/journal.pone.0138566. 26393924 10.1371/journal.pone.0138566 PMC4579124

[34] Meisner M Lohs T Huettemann E The plasma elimination rate and urinary secretion of procalcitonin in patients with normal and impaired renal function. *Eur J Anaesthesiol* 2001; 18:79–87. 11270029 10.1046/j.0265-0215.2000.00783.x

[35] Opatrna S Klaboch J Opatrny K Procalcitonin levels in peritoneal dialysis patients. *Perit Dial Int* 2005; 25:470–472. 16178480

[36] Lu XL Xiao ZH Yang MY Diagnostic value of serum procalcitonin in patients with chronic renal insufficiency: a systematic review and meta-analysis. *Nephrol Dial Transplant* 2013; 28:122–129. 23045429 10.1093/ndt/gfs339

