# Supplementary material for: Impact of hepatic function on serum procalcitonin for the diagnosis of bacterial infections in patients with chronic liver disease: A retrospective analysis of 324 cases
Source: Medicine (Baltimore). 2016 Jul 29;95(30):e4270. doi: 10.1097/MD.0000000000004270 (PMC5265836; doi:10.1097/MD.0000000000004270)
Supplement: Supplemental Digital Content [file medi-95-e4270-s001.doc]

A B

C D

Figure 1S

Figure 1S. Receiver operating characteristic (ROC) curves of procalcitonin (PCT) for predicting infection in patients with impaired liver function.

ROC curves of PCT for prediction of infection in patients with impaired liver function. The area under the curve (AUC) of PCT and best cut–off value were 0.907 (95% CI 0.828–0.958) and 0.38 ng/ml for group A (TBIL<5 mg/dl, A), 0.927 (95% CI 0.844–0.974) and 0.54 ng/ml for Group B (5 mg/dl ≤TBIL<10 mg/dl, B), 0.914 (95% CI 0.820–0.968) and 0.61 ng/ml for Group C (10 mg/dl ≤TBIL<20 mg/dl, C), 0.906 (95% CI 0.826–0.958) and 0.94 ng/ml for Group D (TBIL≥20 mg/dl, D), respectively.
